# Supplementary material for: Integrating Machine Learning and Quantum Circuits for Proton Affinity Predictions
Source: J Chem Theory Comput. 2025 Feb 17;21(5):2235–43. doi: 10.1021/acs.jctc.4c01609 (PMC11912190; doi:10.1021/acs.jctc.4c01609)
Supplement: Supplementary file 1 — ct4c01609_si_001.pdf [file ct4c01609_si_001.pdf]

Supporting Information
Integrating Machine Learning and Quantum Circuits for Proton Affinity Predictions

Hongni Jin[a,b] and Kenneth M. Merz, Jr.[a,b]*

[a]Department of Chemistry, Michigan State University,

East Lansing, Michigan 48824, United States

[b]Center for Computational Life Sciences, Lerner Research Institute,

The Cleveland Clinic, Cleveland, Ohio 44106, United States

*Email: merz@chemistry.msu.edu

Table S1. The optimal hyperparameters of each model.

ML model	ML hyperparameters
SVR	kernel=rbf,<br>C=1000,<br>gamma=0.001
RFR	n_estimators=200,<br>max_depth=70
GBDT	learning_rate=0.05, max_depth=4<br>min_samples_split=7,<br>n_estimators=1000,<br>subsample=0.6
XGBoost	learning_rate=0.1,<br>max_depth=10,<br>n_estimators=1000,<br>colsample_bytree=0.6<br>subsample=0.6
